# “Artificial Wood” Lignocellulosic Membranes: Influence of Kraft Lignin on the Properties and Gas Transport in Tunicate-Based Nanocellulose Composites

**DOI:** 10.3390/membranes11030204

**Published:** 2021-03-13

**Authors:** Ievgen Pylypchuk, Roman Selyanchyn, Tetyana Budnyak, Yadong Zhao, Mikael Lindström, Shigenori Fujikawa, Olena Sevastyanova

**Affiliations:** 1 Division of Wood Chemistry and Pulp Technology, Department of Fiber and Polymer Technology, School of Chemistry, Biotechnology and Health, KTH Royal Institute of Technology, Teknikringen 56-58, 100 44 Stockholm, Sweden; yadong@kth.se (Y.Z.); mil@kth.se (M.L.); olena@kth.se (O.S.); 2 WPI International Institute for Carbon-Neutral Energy Research (WPI-I2CNER) Kyushu University, Ito Campus, 744 Motooka, Nishi-ku, Fukuoka 819-0395, Japan; fujikawa.shigenori.137@m.kyushu-u.ac.jp; 3 Department of Materials and Environmental Chemistry, Stockholm University, Svante Arrhenius väg 16C, 106 91 Stockholm, Sweden; tetyana.budnyak@mmk.su.se; 4 Wallenberg Wood Science Center, Department of Fiber and Polymer Technology, School of Chemistry, Biotechnology and Health, KTH Royal Institute of Technology, Teknikringen 56-58, 100 44 Stockholm, Sweden

**Keywords:** nanocellulose, lignin, nanocomposites, gas separation, biopolymer membrane

## Abstract

Nanocellulose membranes based on tunicate-derived cellulose nanofibers, starch, and ~5% wood-derived lignin were investigated using three different types of lignin. The addition of lignin into cellulose membranes increased the specific surface area (from 5 to ~50 m^2^/g), however the fine porous geometry of the nanocellulose with characteristic pores below 10 nm in diameter remained similar for all membranes. The permeation of H_2_, CO_2_, N_2_, and O_2_ through the membranes was investigated and a characteristic Knudsen diffusion through the membranes was observed at a rate proportional to the inverse of their molecular sizes. Permeability values, however, varied significantly between samples containing different lignins, ranging from several to thousands of barrers (10^−10^ cm^3^ (STP) cm cm^−2^ s^−1^ cmHg^−1^cm), and were related to the observed morphology and lignin distribution inside the membranes. Additionally, the addition of ~5% lignin resulted in a significant increase in tensile strength from 3 GPa to ~6–7 GPa, but did not change thermal properties (glass transition or thermal stability). Overall, the combination of plant-derived lignin as a filler or binder in cellulose–starch composites with a sea-animal derived nanocellulose presents an interesting new approach for the fabrication of membranes from abundant bio-derived materials. Future studies should focus on the optimization of these types of membranes for the selective and fast transport of gases needed for a variety of industrial separation processes.

## 1. Introduction

Cellulose and lignin, the two major components of higher plants, are the most abundant polymers on earth. Nanocellulose is a form of cellulosic material that can be obtained from the smallest organisms (i.e., bacteria) to the largest high plants (i.e., trees). Due to its combination of high mechanical strength and low density, it is expected to be used in a variety of emerging applications, such as in environmentally friendly composite materials, films, coatings, and membranes in the food and pharmaceutical industries and for energy applications [1,2,3]. Taking into their account natural origin, renewable character, and biodegradability, biomass-derived materials are an important research direction, with the aim of substituting the multitude of materials obtained from fossil fuels. The preparations and applications of nanomaterials from biomass are dominated by the use of mechanochemical methods to separate the components followed by processing to provide the materials with specific nanofeatures (e.g., morphology, particle size, or crystallinity) [4,5].

Nanocellulose has considerable potential to extend the applications of conventional cellulose for environmental remediation and filtration purposes (i.e., conventional paper filters) and advanced gas separation (cellulose-derived membranes). The material is versatile and can be used in the form of powders, gels, films, or membranes. For instance, Wei et al. reported nanocellulose and lignin-derived carbon aerogels with a high CO_2_ adsorption capacity of up to 5.23 mmol g^−1^ at 273 K and 100 kPa [6]. Using the combination of graphene oxide, a polymer of intrinsic microporosity (PIM-1), with cellulose acetate, Allamar et al. constructed nanocomposite hydrogels to increase the adsorption of insecticides [7]. In recent work, Zheng et al. combined TEMPO-modified cellulose nanocrystals with oxidized carbon to prepare composite films for selective adsorption of the rare-earth element Dy (III) [8]. Due to its low cost, cellulose can also be used as an affordable support for more advanced membrane fabrication, for example grafting of polymer brushes [9].

Cellulosic materials also have a long history in membranes. Recently, a microfibrillated nanocellulose (MFC) membrane showed outstanding selectivity in the separation of CO_2_ from N_2_ and CH_4_ when a highly humidified gas stream was tested [10]. Bayer et al. [11,12] used different types of nanocellulose in proton exchange membranes (PEM) for fuel cell applications, demonstrating a high gas barrier in dry conditions that was much different from the conventional cellulose-based membranes. Both cellulose nanofiber (CNF) and cellulose nanocrystal (CNC) membranes showed significantly lower gas permeabilities than cellulose membranes. Gas transport rates for small gases (H_2_, CO_2_, N_2_, and O_2_) were more than two orders of magnitude smaller [11] for CNF than for conventional cellulose and state-of-art PEMs (e.g., Nafion). In another application, nanocellulose can be used for filtration membranes similar to conventional paper filters, but with more precise control over the pore sizes. Quellmalz and Mihranyan [13] cross-linked *Cladophora* cellulose with citric acid for size exclusion filters that were able to remove particles as small as 20 nm from a dispersion, showing that nanofiltration applications for virus filtration can be developed, although the structure–property relations in a variety of nanocellulose types need further study.

In plants, cellulose is combined with lignin [14] and hemicelluloses. Lignin, which is also available in large quantities, enhances plant cell wall rigidity and hydrophobic properties and promotes mineral transport through the vascular bundles in plants [15,16]. In the process of paper production from wood, lignin is usually degraded and removed and the cellulose fibers are separated and retained. Once dissolved in cooking liquor, the lignin is generally used as a low-cost fuel, however separation and upgrading, especially of kraft lignin, are now emerging as important topics [17], and the market is expected to reach 3.5–14 Mt/year [18], providing an additional 5–10% revenue for the pulp mills. 

A beneficial synergistic effect of mixing lignin and cellulose in different applications was demonstrated in a review by Balakshin et.al. [17]. The incorporation of lignin as a component of cellulose membranes was reported by Farooq et.al., whereby lignin-containing membranes were applied to absorb UV radiation [19]. Dou et al. reported the manufacture of highly hydrophobic films from willow bark lignocellulose [20], and physisorption studies of CO_2_ capture by lignin-derived carbons have also been reported [6,21,22]. 

Considering the abundant resources of nanocellulose and lignin, which are low-cost and environmentally friendly natural polymers, and the established record of conventional cellulose membranes for certain gas separation applications, the study of their gas transport is of great importance. The understanding of the synergy of the components in recombined formulations is still unclear. In this study, we provide an initial assessment of the gas transport through nanocellulose membranes containing a small fraction (~5 wt%) of a wood-derived kraft lignin. Purified and well-characterized lignins from three different sources (hardwood, softwood) and nanocellulose from tunicate (*Ciona intestinalis*) were used to create materials in which wood-derived lignin was combined with sea-animal derived cellulose. The membranes can, thus, be considered to be a new type of nanocellulose paper within the framework of re-assembled or artificial wood.

## 2. Materials and Methods

### 2.1. Materials

The prehydrolysis–kraft cooking–bleaching method was applied to obtain tunicate cellulose from *Ciona intestinalis* [23]. Enzymatic pretreatment was performed to prepare tunicate CNF, which was adjusted to a concentration of 0.5% *w/w* in water. The degree of polymerization (DP) of the tunicate CNF was 4200 and the crystallinity index was 94%. The charge density of the tunicate CNF was 43 μmol/g, the crystal size was 7.7 nm, and the Iβ ratio was 89.9% [23]. Commercial starch (water-soluble, 80% amylopectin and 20% amylose; Sigma S-9765, Mw: 342.30) was used, without any other treatment.

**Scheme 1:**
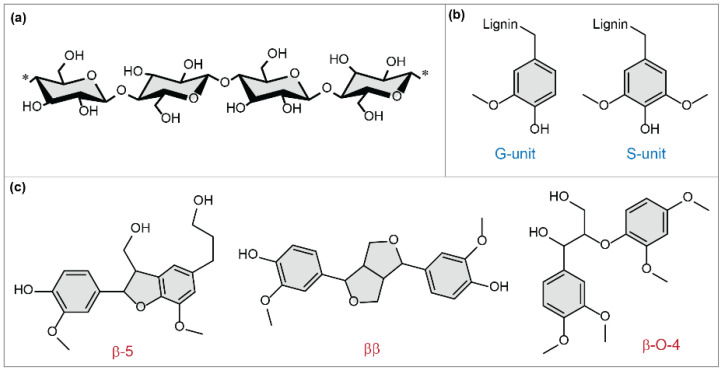
Chemical structures of the cellulose (**a**) and most common molecular units present in lignin: (**b**) main units; (**c**) main interlinkages [27].

Kraft lignin samples from both softwood (Norway Spruce, coded as SW) and hardwood (Rose Gum (Eucalyptus grandis), coded as HW) were obtained using the LignoBoost process [24]. The moisture content of the spruce LignoBoost lignin was 6.4% and that of the eucalyptus LignoBoost lignin was 5.1%. The ash content of the spruce LignoBoost lignin was 0.6% and that of the eucalyptus LignoBoost lignin was 1.2%. Another type of softwood lignin obtained by ceramic membrane ultrafiltration was provided by CleanFlow AB (Sweden, coded as CF lignin). The properties of the HW and SW lignin samples, such as the molecular weight, numbers of phenolic and aliphatic hydroxyls, carboxylic acid content, and polydispersity, were previously reported by Tagami et al. [25]. The properties of the CF lignin were reported by Abbadessa et al. [26]. The chemical structure of cellulose is given in Scheme 1a, while lignin represents a more complex and randomly branched polymer that can be characterized by the presence of characteristic main and interlinkage units, as shown in Scheme 1b,c, respectively [27].

### 2.2. Preparation of Lignin–Tunicate CNF–Starch Membranes

The composite membranes were fabricated by casting a solution of a mixture of lignin, CNF, and starch in high-quality polystyrene Petri dishes with flat, even surfaces. The starch was dissolved in water (0.5 wt%) under continuous stirring at 90 °C to obtain a clear solution, which was cooled to room temperature (sol. 1). Each lignin sample was dissolved in acetone/water (4:1, *v/v*) at a concentration of 0.1 wt.% (sol 2), then 4 mL of the 0.5 wt.% tunicate CNF suspension (sol 3) was blended with 4 mL of the starch solution and 2 mL of the lignin solution, mixed well, directly cast into Petri dishes, and dried in a convection oven at 50 °C overnight to form composite films (referred to as SW/CNF, HW/CNF, and CF/CNF, depending on the type of lignin used). A blank CNF-starch film (referred to as CNF) was made by mixing 4 mL CNF suspension and 4 mL starch solution without lignin, followed by casting and drying [23]. To account for the potential variation of membrane thickness, this was measured for each specific sample and test.

### 2.3. Characterization

The chemical structure of the membranes was analyzed using a scanning FTIR microscope (Nicolet iN10 MX, Thermo Scientific Inc., Waltham, MA, USA) in the range of 4000 to 650 cm^−1^, with a spectral resolution of 4 cm^−1^ used in the attenuated total reflectance (ATR) mode with germanium crystal tip. Each spectrum was averaged from 128 scans and spectral data were analyzed using OMNIC software (Thermo Scientific Inc., Waltham, MA, USA). ATR-FTIR allows non-destructive analysis of membrane samples with several-micron penetration depths. No specific sample preparation was needed, apart from keeping the membrane in a desiccator before the analysis. The pressure was changed accordingly with sample hardness to obtain reliable contact for reproducible spectral acquisitions. 

The morphology of the surface of the membrane was observed using a field-emission scanning electron microscope (JSM-7900F, JEOL, Tokyo, Japan). The samples were coated with a thin platinum layer using an ion sputter instrument (Hitachi E-1030, Tokyo, Japan) before the observations. For cross-section preparation, membranes were broken in liquid nitrogen, attached to the holder using a double-sided conductive copper tape, and sputter-coated with 5 nm of Pt–Pd alloy, before being observed using a field-emission scanning electron microscope (S-4800, Hitachi, Tokyo, Japan). The topographical imaging of the surfaces of the membranes was performed using atomic force microscope (ScanAsyst mode on Bruker Multimode 8 instrument with Nanoscope V controller and E scanner) at room temperature. The membranes were cut into 5 × 5 mm pieces and investigated in the air using a Si-doped antimony cantilever (Bruker RFESP-40 probe).

The mechanical properties of the fabricated membranes were assessed on a single-column Instron 5944 tester (Instron Ltd., Norwood, MA, USA) under ambient conditions (21 °C, 50% humidity). A cell with a 500 N maximum load was used and the pressure of the grips was set to 5 bar. The maximum load was 250 N. The measurements were made on 10 × 5 mm strips. The thickness of each sample was used for the strain calculations. The test was performed on 3 specimens of each lignin-containing sample and once on the reference CNF sample.

The specific surface area and pore size were determined from nitrogen adsorption–desorption isotherms at 77 K (Micromeritics ASAP 2020 sorption analyzer) of samples that had been degassed at 353 K before the measurements. The pore size distribution (PSD) was calculated using the Barret–Joyner–Halenda (BJH) method. 

Thermogravimetric analysis (TGA) was carried out on a TGA/DSC-1 instrument (Mettler Toledo, Columbus, OH, USA) with a heating rate of 10 °C min^−1^, an air flow of 50 mL min^–1^, and a temperature range of 30–600 °C.

Differential scanning calorimetry (DSC) was performed using a Netzsch instrument (DSC 204 F1 Phoenix^®^, Selb, Germany) under a nitrogen atmosphere (flow rate 50 mL min^−1^). The samples weighing 5–6 mg were enclosed in aluminum crucibles and heated from room temperature to well above the glass transition point (T_g_) at 10 °C min^−1^, with an empty crucible used as the reference. The T_g_ value was determined in the second heating run as the midpoint of the onset and the end of a step transition using the Netzsch analysis software (Proteus Analysis). The T_g_ values of the lignins were determined using a Mettler-Toledo DSC 820 instrument (Columbus, OH, USA), with the samples being heated in a 100 µL Al crucible at 10 °C/min, with a N_2_ gas flow of 50 mL min^−1^ up to 220 °C. The T_g_ value was determined in the second heating run as the midpoint value between the onset and the end of a step transition 

**Figure 1:**
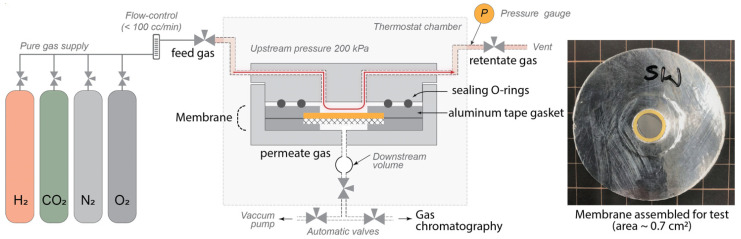
Schematic illustration of the gas permeability measurement setup. Photography shows the membrane prepared for the gas permeability testing—the small membrane area in the middle is exposed to the compressed gas in the GTR-31A system.

For the single-gas permeability measurements, membranes were masked with a metal gasket to provide a circular open area with a diameter of approximately 1 cm, which was stuck to the surface of the membrane using a 5-min two-component epoxy glue (Devcon #14250). The active area of the membrane was precisely measured using image analysis software (ImageJ). The permeation rates of dry gases (H_2_, O_2_, N_2_, CO_2_) through the membranes were measured at 25 °C using the GTR-31A gas barrier testing system (GTR Tec Corp., Japan), in a cell shown schematically in Figure 1. The permeability (P) of the gases in barrer units (1 barrer = 10^−10^ cm^3^ (STP) cm cm^−2^ s^−1^ cmHg^−1^) was estimated for each membrane. The ideal selectivity between two different gases of the composite membrane was calculated as the ratio, α(A/B) = P_A_/P_B_, where P_A_ and P_B_ are the respective permeabilities of gases A and B, respectively.

## 3. Results and Discussion

**Figure 2:**
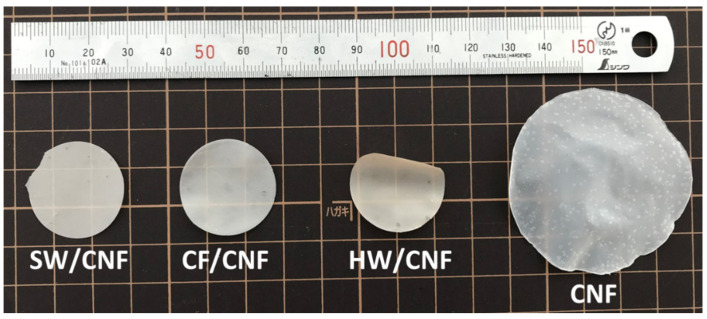
Photograph of the membranes. The lignin-containing samples were more rigid and better attached to the polystyrene dishes used for casting. Only circles 2.5 cm in diameter were recovered, while CNF was completely self-detached from the substrate after drying.

For example, for gas permeability tests, circles measuring 1 cm in diameter were used and the thickness was measured in the precise center of each studied sample. The variability in measured thickness values was not large and changes within several % are considered negligible. Photographs of the nanocellulose membrane with and without different types of lignin are shown in Figure 2. After complete drying of the cast solutions, blank CNF membranes were naturally detached from the polystyrene surface without forceful peeling, whereas samples containing lignin (SW/CNF, HW/CNF, and CF/CNF) were more brittle and could not be easily peeled off. This likely originated from the hydrophobic interaction between lignin and polystyrene both having benzene rings in their structure, which may promote stronger adhesion. To release the membranes, ethanol was added to the Petri dishes, which enabled the membranes to be detached as self-supporting films. The 25-mm-diameter samples shown in Figure 2 were cut using a manual punch (Dumbbell Co. Ltd., Saitama-Ken, Japan). 

**Figure 3:**
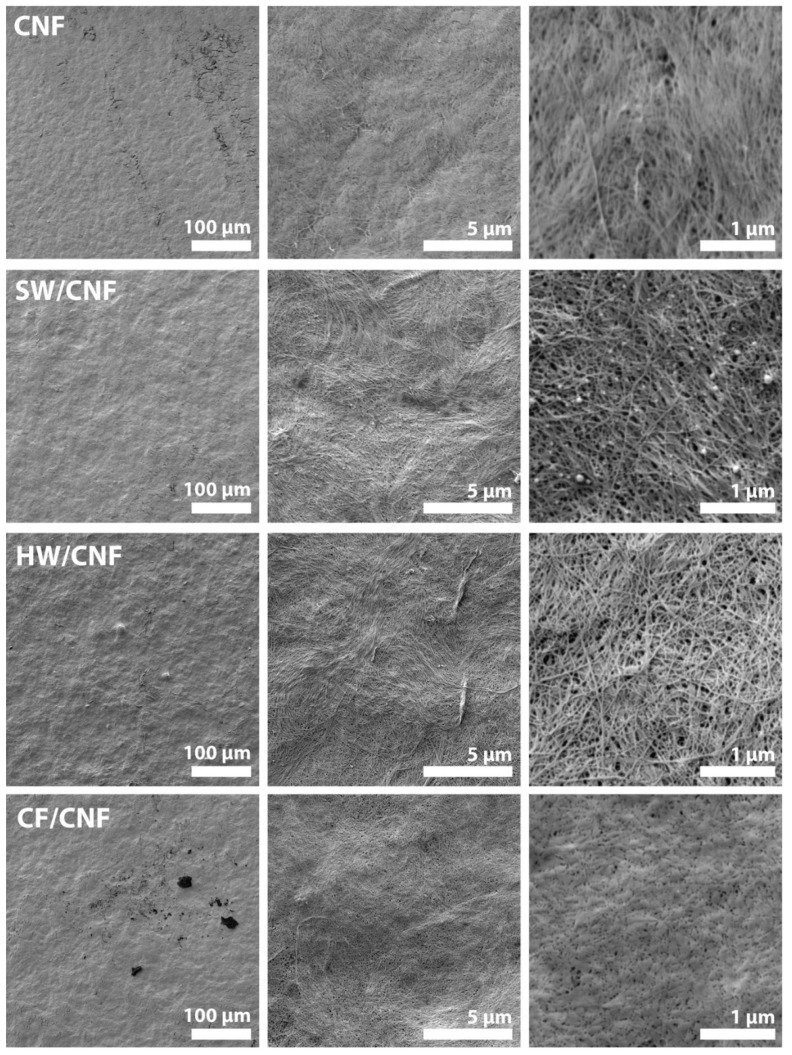
SEM images of surfaces of composite membranes at different magnifications (row 1: CNF, row 2: SW/CNF, row 3: HW/CNF, row 4: CF/CNF).

The membrane surfaces were first investigated using SEM and were found to be uniform, as shown in Figure 3. Observation at higher magnifications revealed that the surfaces of all the membranes were porous, presumably because the high crystallinity of the CNF prevented dense packing of the fibers despite the presence of starch, which was probably uniformly stacked on the fiber surfaces due to the similarity in the chemical structures of these carbohydrates. The addition of lignin into the nanostructure mixed with cellulose nanofibers is believed to modify the porous structure of the nanocellulose with characteristic pores below 10 nanometers. In the case of the SW/CNF membrane (Figure 3, second row), lignin is visible in the form of nanoparticles uniformly mixed with the cellulose nanofibers, whereas in the CF/CNF sample, densification of the surface is apparent (Figure 3, bottom row). 

**Figure 4:**
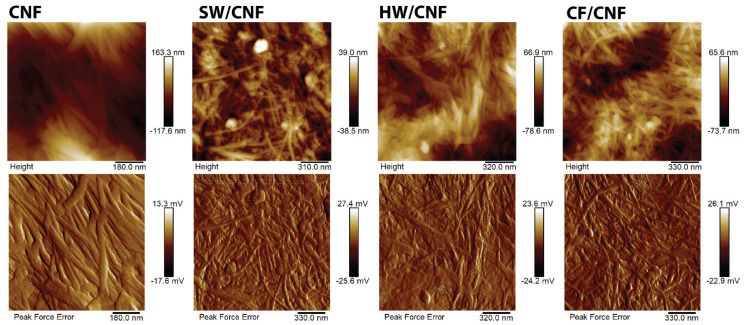
AFM images of the surface of composite lignocellulose membranes: height sensor (**top** row) and peak force error (**bottom** row) images.

To gain better insight into the surface morphology atomic force microscopy (AFM) was used to investigate the topography of the CNF and lignin-containing CNF membranes (Figure 4). The CNF membrane surface was covered by entangled cellulose nanofibrils with a diameter of 27 ± 4 nm. For the lignin-containing membranes, quasi-spherical particles were observed additionally to fibers, which can be attributed to lignin aggregates, and according to the size, to lignin nanoparticles. The largest nanoparticles were observed on the surfaces of SW/CNF membranes, followed by CF/CNF and HW/CNF membranes. The larger nanoparticle size of the SW/CNF membrane can be explained by the higher energy of the interactions between G-units in the structure of softwood lignin than that between S-units [28]. This observation is in line with our previous work, which focused on the formation of lignin nanoparticles, where larger particles for softwood lignin than hardwood lignin were observed [29]. 

**Figure 5:**
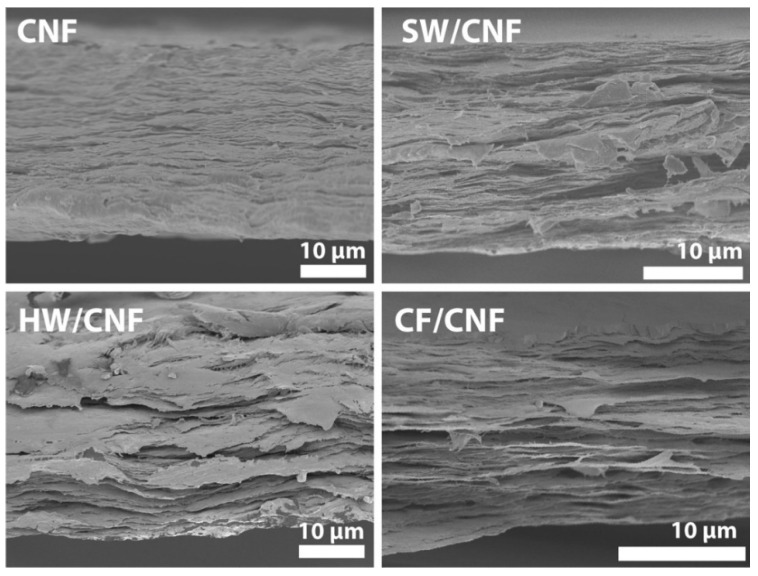
Cross-sectional SEM images of composite lignocellulose membranes.

The cross-sections of the membranes were also studied, and the results are shown in Figure 5. The thicknesses of the membranes were in the range of 20–30 μm. Lignin cannot be distinguished in these images. The SEM images show elongated and intertwined CNF fibrils forming a quasi-layered structure. The simple CNF membrane is packed more densely and more uniformly than the other membranes. The membranes containing softwood lignin (SW/CNF and CF/CNF) have similar surface morphologies, however the HW/CNF membrane surface has a more rough and wavy structure. These characteristic features may be due to electrostatic repulsion between the lignin and cellulose, both of which have a weak, negatively charged surface [30,31]. The morphological differences can also be explained by differences in the energy of interaction in the lignin units (shown in Figure 1). Namely, the main linkages are β-O-4′ substructures, β−5 phenyl coumaran structures, and β−β′ resinol substructures, while the energy of the non-covalent π-π interactions between G-units is higher than that between S-units [28]. This is expected to result in a stronger bonding between softwood lignin molecules (SW and CF) than between hardwood lignin molecules (HW), leading to a denser packing of lignin in the membrane. 

**Figure 6:**
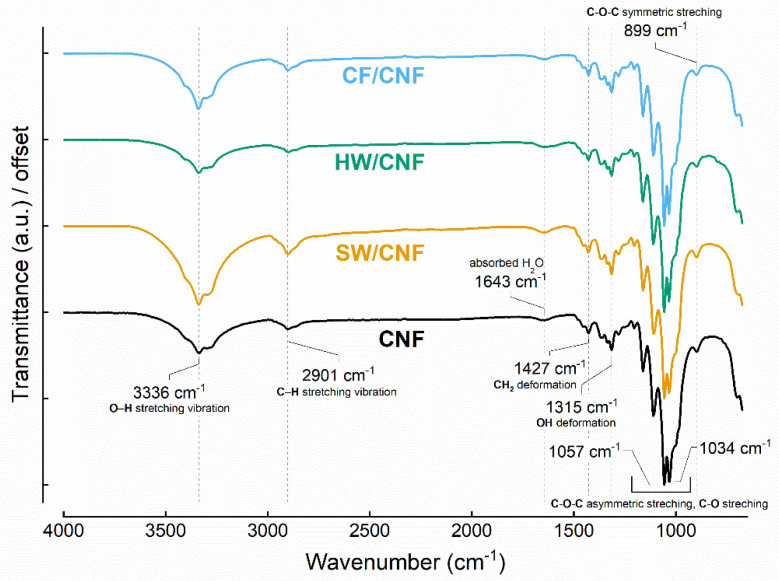
Attenuated total reflection–FTIR spectra of the lignocellulose membranes.

Infrared spectra acquired in the attenuated total reflection mode are shown in Figure 6. The membranes demonstrated similar chemical structures, since they have CNF and starch as the common dominant components (~95%). Adsorption bands around 3300 cm^−1^ and 2900 cm^−1^ can be attributed to the O-H and C-H stretching vibrations, respectively, in the cellulose polymer and starch [32]. They appear as a superposition of sharper bands of highly crystalline nanocellulose with the broad peaks of amorphous starch [33]. The series of peaks in the 1000–1250 cm^−1^ range with the most intense at 1057 cm^−1^ and 1034 cm^−1^ can be attributed to C-O-C vibrations of cellulose pyranose rings. Bands at 1427 cm^−1^ and 1315 cm^−1^ are attributed to the deformation of OH and CH_2_ bonds, respectively, while the broad peak at 1643 cm^−1^ originates from the water adsorbed in the membranes [33]. No absorption bands related to lignin structures are distinguishable in the FTIR spectra due to the low content of lignin in the membranes and the peculiarity of its distribution in the membranes, as demonstrated by AFM.

**Figure 7:**
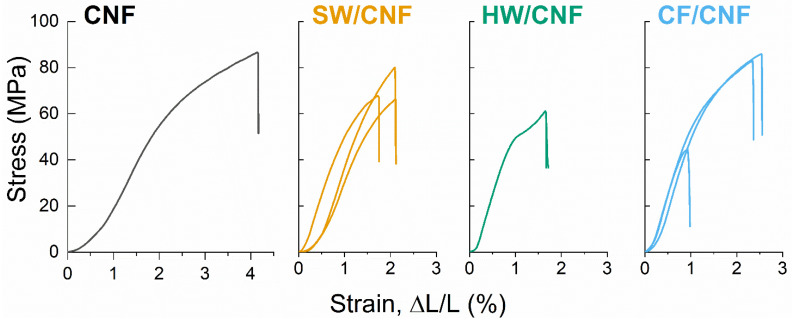
Stress–strain curves for the fabricated lignocellulose membranes.

The mechanical properties were assessed via a tensile test and the results are presented in Figure 7 and Table 1. The addition of ~5% of lignin had a considerable effect on the properties of the membranes, with a ca. two-fold increase in modulus of elasticity from 3.7 to ~6 Gpa and a decrease in the strain at break from ca. 4% to ca. 2% The HW/CNF had the highest modulus and the lowest strain at break The properties of SW/CNF and CF/CNF were similar to each other. Overall, the mechanical tests showed that the lignin acted as a reinforcing component, in line with the results reported in a previous study [23]. The increased modulus is a desired property for the membrane systems in order to achieve better mechanical stability, however flexibility is also usually required, as the membranes need to withstand handling before immobilization in larger module assemblies. For practical applications, the mechanical properties should also be assessed in varying environmental conditions (e.g., various humidity levels).

**Figure 8:**
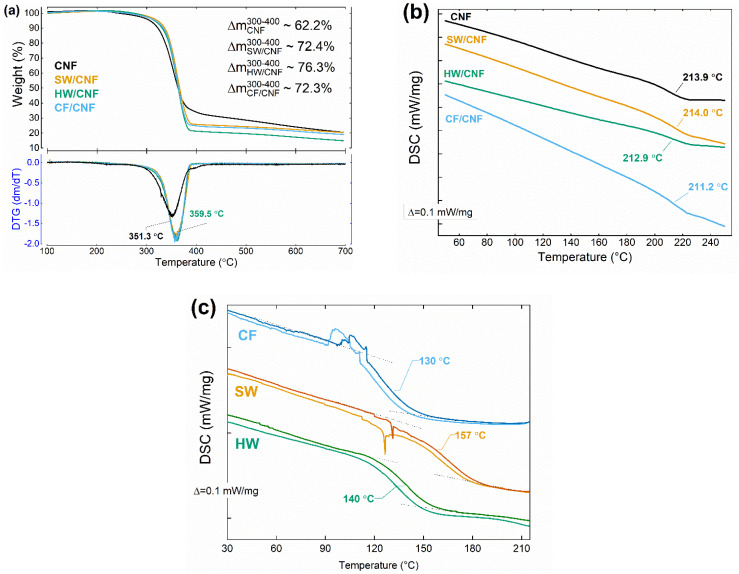
(**a**) Thermogravimetric analysis and (**b**) dynamic scanning calorimetry analysis of the nanocellulose–lignin membranes. (**c**) Dynamic scanning calorimetry analysis of the lignins.

The thermal properties of the CNF membranes were assessed using thermogravimetric analysis. As shown in Figure 8a, all of the membranes demonstrated excellent thermal stability up to 300 °C, when degradation started. The weight loss for the CNF membrane between 300 and 400 °C was 62.2%, however for the SW/CNF, HW/CNF, and CF/CNF composites the values were 72.4%, 76.3%, and 72.3%, respectively. This increase in mass loss was probably due to the decomposition of lignin. According to the differential thermogravimetry (DTG), the pure cellulose membrane had a peak at 351 °C, whereas the lignin-containing membranes had peaks at 359 °C. The fact that the decomposition starts at a higher temperature suggests that the addition of lignin leads to a modest improvement in thermal stability.

The glassy state of the composites was analyzed using dynamic scanning calorimetry (DSC). In Figure 8b, all of the samples show a step-like change in the curve at around 214 °C, indicating that this is the glass transition temperature (T_g_) and that the presence of lignin did not influence this transition. The glass transition is apparently driven by the CNF. The thermal properties of the different lignins are shown in Figure 8c. All of the lignin samples had significantly lower T_g_ values than those of the composites with CNF (130–160 °C), in agreement with the literature [34,35]. The fact that the T_g_ of lignin was not detected in the DSC scans of the composites suggests that it was present in a highly dispersed state, in contrast to its existence as a continuous phase in plant cell secondary walls [14]. 

**Figure 9:**
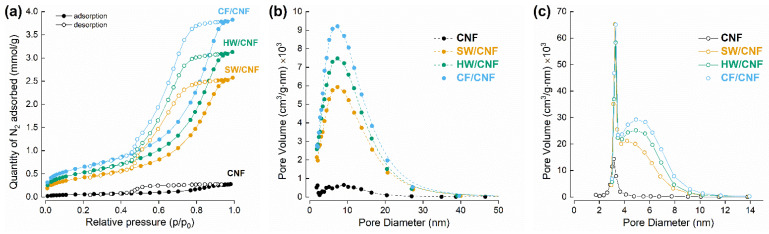
(**a**) Nitrogen adsorption–desorption isotherms and pore-size distribution curves calculated using the Barret–Joyner–Halenda (BJH) method for the (**b**) adsorption and (**c**) desorption branches of the isotherms.

Microscopic investigation of the membranes suggested that they are porous. To verify this quantitatively, nitrogen adsorption–desorption isotherms were acquired for the nanocellulose and lignin-modified materials. The results are provided in Figure 9a—all samples manifested the characteristic type IV isotherms with hysteresis loops with an H2 shape [36,37]. This type of loop is a characteristic feature of mesoporous materials, and it can be also interpreted by percolation effects in highly heterogeneous pore networks [36]. The BET surface area was 4.9 m^2^·g^−1^ for the CNF membrane and 34.7 or 43.7 m^2^·g^−1^ with the addition of softwood or hardwood lignin, respectively. The largest specific surface area (53.0 m^2^·g^−1^) was found for the cellulose membrane with added membrane-filtered CF lignin, indicating that the distribution was more uniform. The total pore volume was found to be 0.089–0.132 cm^3^·g^−1^ for the lignin-containing membranes and 0.009 cm^3^·g^−1^ for the CNF. The pore-size distribution curves in Figure 9b,c were obtained using the Barret–Joyner–Halenda (BJH) method [38]. According to the IUPAC classification [37], all of the synthesized composites were mesoporous materials with pore diameters ranging between 2 and 12 nm according to the desorption branch and between 2 and 40 nm according to the adsorption branch. The characteristics of the materials are summarized in Table 2.

**Figure 10:**
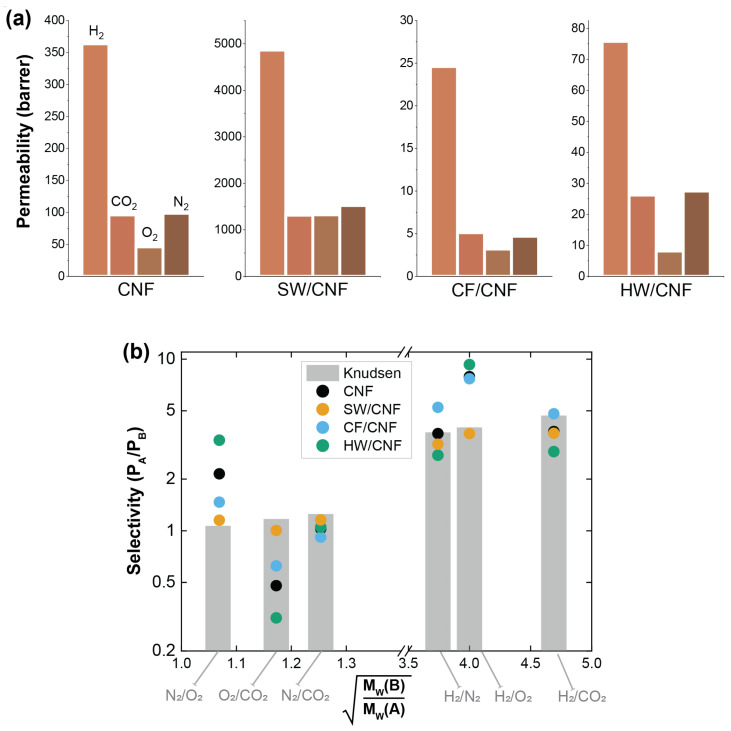
(**a**) Gas permeability through the membranes. (**b**) Selectivity of gas transport in fabricated membranes compared to the Knudsen diffusion model (grey bold line).

The main purpose of this study was to examine the gas transport through the above- described completely bio-based composite membranes. Pure gas permeation was assessed using the differential pressure method. Figure 10 shows the resulting permeabilities for four different gas species through the fabricated membranes. The permeability of hydrogen is greater in all membranes than that of larger molecules. Usually, the permeability of gases through the dense polymeric membranes is described by the solution–diffusion mechanism, while the permeability can be decoupled into the solubility of the gas in the membrane materials and diffusivity. The permeability coefficient can be expressed as a product of solubility and diffusivity coefficients [39]. However, in cases where the membranes are porous, the solubility in and diffusion of gas through the material itself are negligibly small compared to gas transport through the pores. When the pore sizes of a membrane are 0.1 μm or larger, gas permeation takes place by convective flow, as described by Poiseuille’s law [40]. If the pore radius r is much larger than the kinetic diameter of the gas but smaller than the mean free path (λ) of the gas, diffusing gas molecules undergo more collisions with the pore walls rather than with other gas molecules. Gas diffusion in this region is called Knudsen diffusion. On collision with the pore walls, the gas molecules are momentarily adsorbed and then reflected in a random direction. Molecule–molecule collisions are rare, meaning that each gas molecule moves independently of the others. With gas mixtures in which the different species move at different average velocities, separation is possible [40]. In the case of our membranes, if the pores of the membranes were larger, the gases would diffuse at approximately similar rates, without selectivity to certain gas species. Although the selectivities were similar for all the membranes (Figure 10b), the transport rates were very different (Figure 10a). Specifically, the SW/CNF membrane had permeabilities two orders of magnitude higher than those of CNF and three orders higher than those of HW/CNF. Such differences can be explained by the greater porosity, however the adsorption tests showed that it was higher in all of the lignin-containing membranes. The fact that the selectivity was similar in all cases is indirect evidence that all samples had similar pore geometries. Therefore, we suggest that differences in the observed permeabilities were caused by the lignin distribution. Different gas permeabilities correspond well to membrane morphology observations using SEM and AFM; namely, the lignin distributions are different in all samples, ultimately present in the form of larger quasi-spherical particles in SW/CNF, expanding the rigid CNF fiber network. The transport mechanism in the SW/CNF membrane is closest to Knudsen diffusion (orange circles on the selectivity plot close to the Knudsen grey bars), which can be explained by the differences in molecular weights of different gas species (x-axis in Figure 10b). 

**Figure 11:**
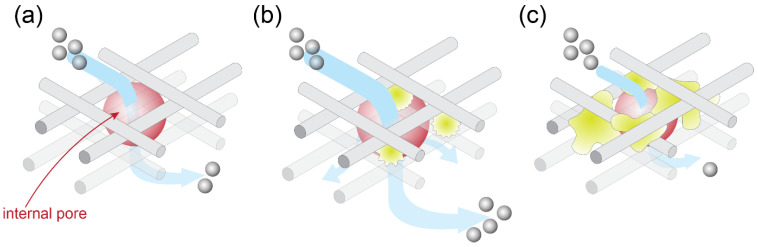
Distribution of lignin inside the CNF matrix, which could explain the observed porosity and gas permeability data (**a**) for the blank CNF–starch network. (**b**) CNF network expanded with weakly interacting lignin particles (yellow; SW/CNF). (**c**) CNF network with strongly interacting lignin molecules covering the fibers and able to disrupt the percolated pathways needed for gas transport but not changing the overall pore structure.

We can suggest that the variation in gas permeabilities was strongly influenced not only by the amount but also by the distribution of the lignin in the composites. The suggested difference is shown schematically in Figure 11. The chemical nature of the lignin determines how it interacts with the cellulose–starch matrix, expanding the CNF network with SW/CNF or creating a micromorphology impeding the gas transport in CF/CNF. Taking into account the morphology and porosity of the membranes, the gas permeation should be considered as a geometrical rather than a chemical problem. This has been very well studied in the literature; however, gas flow description is rather complex [41,42]. A recent numerical simulation study of permeability in fibrous–particle structures with the application of fractal models showed that permeability is expected to increase with an increase of particle diameter [42]. This outcome corresponds to the observed results of the SW/CNF membrane, in which SW lignin is indeed present in the form of quasi-spherical particles. In cases where lignin takes up more random shapes, the numerical description would be even more complex. 

The important result of the gas transport study is that the fabricated membranes fit into the intermediary range of porosity between dense membranes (where gas can be separated via size-sieving or solution–diffusion mechanism) and ordinary Poiseuille law-governed flow membranes (non-selective). The achieved selectivities of such membranes are, however, too low for any practical gas separation applications. However, the result is relevant because there is a strong interest in utilizing widely available biopolymers such as cellulose and lignin in a variety of applications. In respect to gas permeability, nanocellulose is often mentioned as a good gas barrier material, and therefore is suggested to be used for sustainable packaging or other applications [1,3,10,11,12,43,44,45]. The results of our work show that this cannot be a default assumption, as the gas barrier will strongly depend on the material micromorphology and crystallinity. To put the results of the obtained permeabilities into context, they are compared with several reports from the literature in the Table A1 of the Appendix A. The gas permeabilities in the presented membranes were much larger compared to dense membranes. While some studies reported highly selective gas transport through nanocellulose-based membranes [10,44], the permeabilities of dry membranes are usually extremely low, making such membranes impractical for gas separation. In this respect, we reported nanocellulose membranes to demonstrate trade-off phenomena between selectivity and permeability [46]. At the same time, other uses of nanocellulose were found for the microfiltration of nanoparticles (significantly bigger than gas molecules) [13]. Gas transport in the membranes presented in this work fitted roughly in between these two extreme cases, and although some selectivity was observed due to the Knudsen mechanism, this is not practical for the actual gas separation applications. As the studies of gas permeability in nanocellulose-based membranes are still scarce, we believe that this work will indicate to other researchers that when highly crystalline nanocellulose fibers are used, they are unlikely to create the dense structure usually needed to achieve practical differences in gas transport rates, and as a result the good separation ability. However, these types of highly crystalline mechanically strong fibers with the addition of lignin can be used as reinforcing elements in various composites.

## 4. Conclusions

The current study presented an initial investigation of gas transport along with a thorough characterization of a new class of completely bio-based membranes composed of tunicate nanocellulose, starch, and wood-derived lignin. The composites that can be referred to as “artificial wood” recombine the structures of materials commonly found in higher plants but having different, non-plant-based origins. The addition of 5% lignin to CNF membranes led to significant changes in the properties, depending on the chemical structure, botanical origin, and polydispersity of the lignin. The improved mechanical strength of membranes containing lignins was shown by the higher elastic modulus of ~ 6 GPa in lignin-containing membranes compared to ~ 3 GPa in blank CNF. The porosity of the membranes and specific surface areas studied using nitrogen adsorption increased in all samples containing lignin, however no significant differences were observed in pore geometries. Microscopy studies (using SEM and AFM) showed that lignins are present in the membranes in the form of dispersed nanoparticles. 

The investigation of the gas permeabilities through the CNF and CNF–lignin membranes surprisingly showed very different gas permeabilities (e.g., CO_2_ permeability values of 1315, 26, and 5 barrers in lignin–CNF composites vs. 96 barrers in blank CNF). This suggests the importance of molecular interactions between lignin and cellulose and the resulting composite structure morphology, which can provide higher or lower permeabilities depending on the composition. Gas selectivities indicate a Knudsen mechanism of permeation in all membranes, with larger selectivity for hydrogen as the gas with the smallest kinetic diameter.

This study highlights that in the engineered membranes, the morphologies and properties will strongly depend on the components, and therefore may bring the level of control needed for environmentally friendly materials completely made of natural sources. However, future studies should be focused on lignin–cellulose combinations forming denser structures or ultramicroporous membranes, which are expected to result in higher selectivity towards important gas separations.

## Figures and Tables

**Table 1:** Mechanical properties of the fabricated films.

Sample	Modulus (MPa) ^a^	Tensile Strain at Break (%) ^a^	Tensile Stress at Break (MPa) ^a^
CNF	3771	4.1	86
SW/CNF	5670 ± 530	1.98 ± 0.217	71.3 ± 7.6
HW/CNF	6785 ± 6	1.65 ± 0.001	61.2 ± 0.0
CF/CNF	6677 ± 175	2.45 ± 0.142	84.4 ± 2.0

Note: ^a^ average value ± SD, excluding outliers.

**Table 2:** Textural characteristics of materials.

Sample	*S*_BET_, m^2^/g	*V*_pores_, cm^3^/g	*D*_pores_ (ads), nm	*D*_pores_ (des), nm
CNF	4.96 ± 0.14	0.009	8.1	3.3
SW/CNF	34.7 ± 0.16	0.089	8.3	4.6
HW/CNF	43.7 ± 0.27	0.108	8.0	4.9
CF/CNF	53.0 ± 0.22	0.132	8.1	5.0

Note: *S*_BET_—the Brunauer–Emmet–Teller (BET) surface area; *V*_pores_—total pore volume; *D*_pores_—BJH average pore diameter calculated from adsorption and desorption branches of isotherms.

## Appendix A

**Table A1:** Comparison of the gas permeability values of the developed lignin–CNF membranes with examples from the literature.

Membrane Description	Preparation Method	Thickness, µm	Gas Permeability Test	Permeability, Barrer (± SD Where Available)				Ref.
				CO_2_	N_2_	O_2_	H_2_	
Cellulose whiskers (W) from native sisal fibers	casting aqueous solutions in Teflon molds, evaporation at 25 °C, 5 days	~20	CVVP ^a^, Δp = 1 bar	118.8 ± 1.2	161.7 ± 3.2	140.7 ± 2.7	—	[44]
Microfibrillated cellulose (MFC) from native sisal fibers				0.100 ± 0.002	0.150 ± 0.004	0.090 ± 0.001	—	
TEMPO-oxidized cellulose nanofibrils with free carboxyl groups (TOCN-COOH)	casting aqueous dispersions of 0.1% (*w/v*) on PET film followed by drying	~13	Differential pressure ^b^	2.02 × 10^−6^	7.07 × 10^−8^	4.95 × 10^−7^	2.41 × 10^−5^	[45]
TEMPO-oxidized cellulose nanofibrils with sodium carboxylate groups (TOCN-COONa)		~13		2.03 × 10^−6^	1.18 × 10^−7^	8.57 × 10^−7^	2.46 × 10^−4^	
Cellulose nanocrystals (CNC)	Vacuum filtration followed by hot-pressing for 20 min (at 110 °C and 1.1 MPa)	~30	Differential pressure ^b^, Δp = 2 bar, r.t.	—	—	—	1.33 × 10^−2^	[12]
Cellulose nanofibers (CNF)		~30		—	—	—	3.80 × 10^−2^	
Sulfonated cellulose nanofibers (CNF)		~30		—	—	—	1.41 × 10^−1^	
Carboxymethyl cellulose (CMC)	casting aqueous dispersions of 2% on stainless-steel substrate followed by vacuum drying	n/a	CVVP ^a^, pure gases, Δp = 1 bar ^a^	2.50 × 10^−5^	1.02 × 10^−5^	—	1.48 × 10^−4^	[46]
CNF (tunicate)	casting aqueous dispersions of 0.5% (*w/v*) in PS molds followed by drying	37	Differential pressure ^b^, Δp = 2 bar, r.t.	96 ± 0.7	98.7 ± 0.9	46 ± 4.9	363.5 ± 12.5	This work
SW/CNF		22		1315 ± 6	1523 ± 7	1321 ± 19	4864 ± 45	
HW/CNF		22		26.2 ± 0.4	27.5 ± 0.4	8.2 ± 0.8	75.8 ± 3.4	
CF/CNF		24		5.1 ± 0.2	4.7 ± 0.4	3.2 ± 0.4	24.6 ± 1.3	

^a^ CVVP - constant volume, variable pressure ("time-lag") method; ^b^ Differential pressure method according to JISK7126A and ASTMD1434 standards.
